# Epidemiology of bloodstream infections in patients with acute myeloid leukemia undergoing levofloxacin prophylaxis

**DOI:** 10.1186/1471-2334-13-563

**Published:** 2013-12-01

**Authors:** Francesco Giuseppe De Rosa, Ilaria Motta, Ernesta Audisio, Chiara Frairia, Alessandro Busca, Giovanni Di Perri, Filippo Marmont

**Affiliations:** 1 Department of Medical Sciences, Infectious Diseases, Amedeo di Savoia Hospital, University of Turin, Corso Svizzera 164, 10149 Turin, Italy; 2 SC Haematology II, AOU Città della Salute e della Scienza, San Giovanni Battista Molinette Hospital, Turin, Italy

**Keywords:** Acute myeloid leukemia, Neutropenia, Levofloxacin prophylaxis, Febrile neutropenia, Chemotherapy, Infections

## Abstract

**Background:**

Infections are a common cause of morbidity and mortality in patients with acute myeloid leukemia (AML). The evidence for efficacy of antibiotic prophylaxis in reducing the mortality rates and the incidence of bacterial infections was also reported by a systematic review published by Cochrane in 2012. The objective of our study was to report the incidence and the etiology of bloodstream infections in patients with AML undergoing levofloxacin prophylaxis during neutropenic episodes.

**Methods:**

This was a retrospective study of patients with diagnosis of AML during 2001–2007.

**Results:**

A total of 81 patients were included in the study. Two hundred and ninetyone neutropenic episodes were studied, of which 181 were febrile. Bacteria isolated from blood cultures were mostly Gram-positives during the induction (80%) and Gram-negatives during the consolidation (72.4%) phases of chemotherapy. Resistance to ciprofloxacin was found in 78.9% of isolated *E. coli* and it was higher during consolidation and higher than the hospital rate. The production of extended spectrum betalactamases (ESBL) in *E. coli* strains was reported in 12.1%, below the reported hospital rate during the study period.

**Conclusions:**

Regular microbiology surveillance is needed to better understand the impact of levofloxacin prophylaxis in neutropenic patients. Our study shows that Gram-positive bacteria are predominant during the induction phase of chemotherapy and Gram-negatives during the consolidation. The rate of fluoroquinolone resistance in the latter setting, even higher than the hospital rate, may suggest to reconsider levofloxacin prophylaxis.

## Background

Acute myeloid leukemia (AML) is the most common acute leukemia in adults. Standard treatment is conventionally divided into the induction phase with anthracycline and cytarabine and consolidation therapy consisting of cycles of chemotherapy or stem cell transplantation [1,2].

The survival rate is influenced by the prevention and management of infectious complications. Infections are a common cause of morbidity and mortality in patients with AML. The risk-assessment for infections in neutropenic patients, according to the IDSA guidelines is classically divided into high risk (prolonged neutropenia, >7 days; neutrophils count ≤ 100/mm^3^; substantial concurrent comorbidity; clinically unstable) and low risk (neutropenia expected to resolve within 7 days; no active medical comorbidity, clinical stability at onset of the febrile episode; most of them are patients with solid tumors receiving conventional therapy) [3,4].

Levofloxacin prophylaxis during neutropenia for high risk patients has been shown to be effective to prevent all infection related events by a study published in 2005 by GIMEMA group and confirmed by a Cochrane systematic review in 2012 [5,6]. However, empirical antifungal therapy and investigation for invasive fungal infections should be considered for patients with persistent or recurrent fever after 4–7 days of antibiotics and whose duration of neutropenia is expected to be >7 days [7]. Preemptive antifungal management is acceptable as an alternative to empirical antifungal therapy in a subset of high-risk neutropenic patients [8,9].

The primary objective of this study was to report the incidence of fever and clinically or microbiologically proven bacterial or fungal infections during neutropenic episodes of patients with AML undergoing levofloxacin prophylaxis. Our focus was on bloodstream infections [10].

## Methods

This retrospective analysis was conducted in the Department of Hematology 2, at San Giovanni Battista Hospital in Turin between June 2001 and December 2007. The Ethics Committee approval was unnecessary due to the retrospective nature of the study and was waived with the approval of the Hospital Medical Direction for patients given prophylaxis with levofloxacin before 2006. Beginning in 2006, consecutive adult patients with AML were prospectively included in the multicenter AML 02/06 (EudraCT number 2006-003817-429) study by our center, belonging to the Northern Italy Leukaemia Group (NILG) and approved by the Ethical Committee. All included patients were treated with standard induction chemotherapy ICE followed by post-remissional therapy with repeated consolidation courses with high dose cytarabine (HDAraC) plus peripheral progenitor cell support [11]. Each patient signed an informed consent to be included in the study and to receive chemotherapy, antinfective and nutritional therapy. As prescribed in the protocol, all patients received antibiotic prophylaxis with oral levofloxacin at 500 mg/die, given during the induction phase from the day of hospitalization until the recovery of blood neutrophils count over 1000/μl, after chemotherapy. In the following courses of chemotherapy levofloxacin at the same dosage was administered from first day after the end of chemotherapy until the recovery of blood neutrophils count over 1000/μl. Antifungal prophylaxis was administered to all patients with oral itraconazole 200 mg twice a day.

In patients with neutropenia (≤ 500/mm^3^) and fever (defined as external temperature ≥ 38°C) the following baseline diagnostic investigations were performed: chest X-rays, blood cultures, sputum and urine cultures, serum galactomannan antigen, *Streptococcus pneumoniae* and *Legionella* spp. urinary antigens. Empiric treatment, based on international recommendation at the time, could vary among the centres, according to local epidemiology. Neutropenic febrile patients were treated with empirical antibiotic therapy with piperacillin-tazobactam or meropenem. Vancomycin and/or amikacin were added if fever was deemed to be complicated, such as suggestion of intravascular catheter-related infection, MRSA colonization, hypotension and/or organ failure.

Data collected included demographics, presence and characteristics of central venous catheter (type, site, insertion and removal), duration of antibiotic prophylaxis received, description of febrile episodes (duration, initial neutrophil count, blood pressure, SO2%, respiratory rate, body temperature), type and length of antibiotics, results of investigations (chest X-ray and CT scan, ultrasounds, brain CT scan) and microbiology tests (site of infection, analyzed material, isolation of microorganism).

Data was entered into an electronic database and analyzed with Microsoft Excel. Statistical analysis was performed with STATA 11 program (Stata Corporation, USA). Chi-square test was used for categorical variables. Continuous variables were compared by Student’s t-test if normally distributed and the Mann–Whitney U-test if non-normally distributed. All *p* values were two sided, *p* value of <0.05 was considered significant. Values for continuous and categorical variables are expressed as the mean ± SD and median (IQR) or percentage of the group from which they are derived, respectively.

## Results

A total of 81 patients with diagnosis of AML were observed during the study period (46 males and 35 females). The median age was 49.7 ± 11.4 years (range 23–69 years). There were 291 neutropenic episodes, 81 (27,8%) during in the induction and 210 (72,2%) during the consolidation phase; fever was recorded in 181 episodes, 69 during induction (85.2%) and 112 (53.4%) during consolidation cycles. Of the latter 112 episodes 32 (28,6%) were during the second cycle; 25 (22,3%), 27 (24,1%), 17 (15,2%) and 11 (9,8%) during the third, fourth, fifth and sixth consolidation cycle, respectively. The characteristics are reported in Table 1. The mean duration of neutropenia was 14 (range 13–19) days for the induction phase (ICE), 7 (range 5–11) and 5 (range 4–7) days for the second and the third consolidation cycle, respectively. The median duration of neutropenia following the peripheral stem cells transplantation was 12 days. The median number of days of fever was significantly higher in the induction phase than in the consolidation chemotherapy (9 vs 4 days, *p* < 0.001).

**Table 1:** Comparison of neutropenic episodes according to clinical or microbiological confirmation of infection

	**Induction**	**Consolidation**	***p *** **value**
	**(N = 81)**	**(N = 210)**	
Fever	27 (33,4%)	40 (19,1%)	< 0.001
Clinical diagnosis	9 (11,1%)	5 (2,4%)	0.002
Microbiological diagnosis	24 (29,6%)	66 (31,4%)	0.766
Possible IFI	2 (2,5%)		
Probable IFI	3 (3,%)		
Definite IFI	4 (4,9%)	1 (0,5%)	< 0.001
**Total**	**69**	**112**	
Patients without fever	12 (14,8%)	98 (46.6%)	< 0.001

All the patients carried a Hohn central venous catheter both in induction and in consolidation phase. Twenty-four patients underwent allogenic transplantation (29,6%). In twenty-six patients (32,1%) high-dose of citarabine was followed by peripheral stem cells transplantation (preceded by rescue of peripheral blood progenitor cells).

Pneumonia (fungi included) was the most common clinical manifestation: during the induction chemotherapy in 69 patients with fever (19 cases, 27,5%) and during the consolidation chemotherapy in 112 febrile neutropenia episodes (12 cases, 10,7%).

Piperacillin/tazobactam was mainly administered as empirical treatment, followed by meropenem or ceftazidime in association with amikacin; vancomycin was empirically added in 21, 11 and 4 cases, respectively. Resolution of fever during induction chemotherapy was observed in 47,6% of patients treated with piperacillin/tazobactam, in 42,9% with ceftazidime and amikacin and 40% with meropenem. A higher rate of clinical success (disappearance of fever or resolution of clinical manifestation) was reported during the consolidation phase in 91,7% of patients treated with meropenem, 88,9% treated with meropenem and glicopeptide, 75% treated with ceftriaxone and in 68,7% of those who received ceftazidime and amikacin. The lower duration of neutropenia during the consolidation probably explained the higher rate of fever disappearance in the consolidation phase compared with the induction phase (79.5% versus 52.2%, *p* = 0.00018).

Amongst the febrile neutropenic episodes, 29% were associated with bacteraemia during the induction (20 positive blood cultures for bacteria over 69 febrile neutropenic episodes) and 51% during the consolidation phase (59 positive blood cultures for bacteria over 112 febrile neutropenic episodes).

Amongst bloodstream isolates, all coagulase-negative Staphylococci were methicillin-resistant and resistant to fluoroquinolones. *Enterococcus faecalis* isolates were resistant to ampicillin but sensitive to vancomycin and teicoplanin. Amongst *Enterococcus faecium*, vancomycin and teicoplanin were active *in vitro* against two out of three isolates (66,6%). The resistance to fluoroquinolones was also complete amongst the strains of *Corynebacterium spp* and it was 78% in *Escherichia coli*, which were ESBL producers in 12.1% of cases (N = 4), all in the consolidation phases.

The main finding of this study was that bloodstream isolates were predominantly represented by Gram-positives, 80% vs 27,6% (16 cases out of 20 vs 17 cases out of 59) or by Gram-negatives, 20% vs 72,4% (4 cases out of 20 vs 42 cases out of 59) during the induction or consolidation phases, respectively (*p* < 0.001) (Table 2).

**Table 2:** Etiology of positive blood cultures during the induction and consolidation phase

	**Gram-positives**	**Gram-negatives**
	**(N = 33)**	**(N = 46)**
**Induction (N = 81)**	*S. epidermidis* (11)	*E. cloacae* (2)
**Neutropenic fever episodes = 69**	*E. faecium* (3)	*E. coli* (1)
	*S. mitis* (1)	*K. pneumoniae* (1)
	*Corynebacterium* spp. (1)	
**Total (N = 20)**	**16**	**4**
**Consolidation (N = 210)**	*S. epidermidis* (8)	*E. coli* (32)
**Neutropenic fever episodes = 112**	MRSA (3)	*P. aeruginosa* (3)
	*S. hominis* (3)	*E. cloacae* (2)
	*Corynebacterium* spp (2)	*S. maltophilia* (2)
	*E. faecalis* (1)	*S. marcescens* (1)
		*Citrobacter* (1)
		*K. pneumoniae* (1)
**Total (N = 59)**	**17**	**42**

There were ten cases of fungal infections, all but one during the induction phase (2 possible, 3 probable and 5 proven). Proven invasive pulmonary aspergillosis were diagnosed in four cases.

During the study six patients died (7,4%): four with invasive fungal disease, one with Legionella pneumonia and refractory disease and one with refractory disease without signs of infection.

## Discussion

Levofloxacin prophylaxis of febrile episodes in neutropenic patients has been shown to be effective by the GIMEMA study in 2005 [5]. In the same paper the authors also emphasized the need for surveillance for the possible development of antibiotic resistance. In the following years both IDSA guidelines [3] and a Cochrane review of 109 randomized trials [6] confirmed the utility of levofloxacin prophylaxis in selected patient populations with oncohaematologic disease. In this study we retrospectively evaluated neutropenic episodes in patients with AML who received levofloxacin prophylaxis. We found a statistically significant prevalence of Gram-positive bacteria during induction chemotherapy and Gram-negatives during consolidation phase.

The use of prophylactic levofloxacin during every cycle of chemotherapy may have a role in selection of isolated strains, as suggested by Bucaneve *et al*. in their seminal paper. A recent systematic review of RCTs and quasi-RCTs by Cochrane in 2012 demonstrated that levofloxacin prophylaxis significantly reduced all-cause mortality and infection-related mortality, compared to placebo and also significantly reduced febrile episodes [6]. According to those results, quinolone prophylaxis did not increase the incidence of Gram-positive bacteraemia and, more importantly, there was no significant differences in the number of patients developing infections caused by organisms resistant to quinolones.

The spectrum of coverage of fluoroquinolones may account for a lower incidence of bacteraemia by Gram-negatives in the induction phase observed in our study, where the isolation of Gram-positive bacteria may be explained by partial efficacy of the prophylaxis regimen, excluding multidrug resistant strains. The significantly higher prevalence of Gram-negatives during the consolidation phase may be explained by the previous administration of levofloxacin prophylaxis, since as many as 73,7% of Gram-negatives were resistant to fluoroquinolones (78.9% of *E. coli* were resistant to ciprofloxacin), corresponding to 59,6% of the total number of Gram-positives and Gram-negatives. In the induction phase Gram-negatives bacteria resistant to fluoroquinolones accounted for only 15,4% of the total number of bacteria.

Of course we cannot exclude that differences in etiology could be due to other factors, such as comorbidities and hospital stay. However, during the study period there was a lower rate of fluoroquinolones resistance amongst bloodstream *E. coli* hospital isolates (29-38%) and there were few cases of bloodstream infections caused by ESBL-producers *E. coli*, with resistance to fluoroquinolones in 76.3% of strains [12]. Amongst patients undergoing allogenic transplant and levofloxacin prophylaxis, coagulase-negative staphylococci were the most commonly isolated Gram-positive pathogens and *E. coli* was the most commonly isolated Gram-negative bacteria [13]. Resistance to fluoroquinolones was 87% amongst Gram-positive isolates and 50% amongst Gram-negative rods. The issue of growing rate of fluoroquinolone resistance and ESBL production by Gram-negatives may require special attention even at hospital admission in selected patient population where a clinical risk score may be useful [14].

However, since fever disappeared more rapidly during the consolidation phases of chemotherapy, because of reduced duration of neutropenia together with the probable effect of empiric antibiotic treatment, the administration of levofloxacin prophylaxis might be discussed under the view of the local epidemiology, especially when multiple or prolonged administration is expected.

## Conclusions

Fluoroquinolone prophylaxis is frequently used worldwide in high-risk neutropenic patients. However, few reports have studied the rate of resistance amongst Gram-positive and Gram-negative bloodstream isolates during induction and consolidation phases. In our study the rate and resistance of Gram-negatives was significantly higher during the consolidation phase, where the duration of neutropenia is lower and the resolution of fever after empiric antibiotic treatment is higher. If these data will be confirmed, levofloxacin prophylaxis might be at least less extensively administered during the consolidation chemotherapy. Constant monitoring for fluoroquinolone resistance amongst Gram-negative bacteria is required to preserve the efficacy of levofloxacin prophylaxis.

## Abbreviations

AML: Acute myeloid leukemia; ESBL: Extended spectrum beta lactamases; IFI: Invasive fungal infection.

## Competing interests

The authors declare that they have no competing interests.

## Authors’ contributions

FDR, FM, EA contributed to study design, data collection, interpretation of data and statistical analysis. CF contributed to data collection. FDR, IM, FM, EA drafted the first version of the manuscript and finalized the manuscript. AB, GDP contributed to study design, supervision and critical revision of the manuscript for intellectual content. All authors read and approved the final manuscript.

## Pre-publication history

The pre-publication history for this paper can be accessed here:

http://www.biomedcentral.com/1471-2334/13/563/prepub
